# Quantifying the concentration dependence of sedimentation coefficients for globular macromolecules: a continuing age-old problem

**DOI:** 10.1007/s12551-021-00793-x

**Published:** 2021-04-10

**Authors:** Donald J. Winzor, Vlad Dinu, David J. Scott, Stephen E. Harding

**Affiliations:** 1 School of Chemistry and Molecular Biosciences, University of Queensland, Brisbane, Queensland 4072 Australia; 2 National Centre for Macromolecular Hydrodynamics, School of Biosciences, University of Nottingham, Sutton Bonington, LE12 5RD UK; 3 Research Complex at Harwell, Rutherford Appleton Laboratory, Oxfordshire, OX11 0FA UK; 4 University of Oslo, Kulturhistorisk museum, Frederiks gate 2, Oslo, 0164 Norway

**Keywords:** Concentration dependence, Sedimentation coefficient, Sedimentation velocity, Ultracentrifugation, Optical registration

## Abstract

This retrospective investigation has established that the early theoretical attempts to directly incorporate the consequences of radial dilution into expressions for variation of the sedimentation coefficient as a function of the loading concentration in sedimentation velocity experiments require concentration distributions exhibiting far greater precision than that achieved by the optical systems of past and current analytical ultracentrifuges. In terms of current methods of sedimentation coefficient measurement, until such improvement is made, the simplest procedure for quantifying linear *s*-*c* dependence (or linear concentration dependence of 1/*s*) for dilute systems therefore entails consideration of the sedimentation coefficient obtained by standard *c*(*s*), g*(*s*) or *G*(*s*) analysis) as an average parameter ($\overline{s}$) that pertains to the corresponding mean plateau concentration (following radial dilution) ($\overline{c}$) over the range of sedimentation velocity distributions used for the determination of $\overline{s}$. The relation of this with current descriptions of the concentration dependence of the sedimentation and translational diffusion coefficients is considered, together with a suggestion for the necessary improvement in the optical system.

## Introduction

The problem of allowing quantitatively for concentration dependence of the sedimentation coefficient for globular proteins and macromolecular assemblies has been an issue for ultracentrifuge chemists for eight decades. This problem extends to the translational diffusion coefficient which can also be measured by the analytical ultracentrifuge (along with other methods such as dynamic light scattering). Traditional methods of extrapolating weighted average sedimentation coefficients to zero concentration to eliminate the effects of hydrodynamic non-ideality (through co-exclusion, charge repulsion, solvation and back-flow effects of solvent) become invalid when associative effects (self-association A-A, A-A-A... and heterologous A-B, A-A-B, A-B-C…, etc interactions) are being explored and, in particular, quantified in terms of association/dissociation constants (popularly, for A-A and A-B systems the “dissociation constant” *K*_d_, expressed in μM). Progress has nonetheless been made at least under dilute solution conditions, largely based on rigid-sphere theory (see for example Burgers 1941a,b, 1942a, b; Pyun and Fixman 1964; Batchelor 1972; Beenakker and Mazur 1983; Harding and Johnson 1985a; Brady and Durlofsky 1988; Cichocki and Felderhof 1990; Hayakawa and Ichiki 1995); and a consensus is slowly emerging.

A further complication — often ignored — is that experimental quantification still causes difficulties because of uncertainty about the concentration that should be ascribed to a measured sedimentation coefficient, *s*. This uncertainty stems from the “radial dilution effect”, an effect that has been known about for decades (Schachman 1959). Account needs to be taken of the consequences of an ever-decreasing solute concentration as the result of radial dilution in a sector-shaped cell—a situation at variance with the assumption of concentration-independent migration that is inherent in the traditional procedure for sedimentation coefficient measurement (Svedberg and Pedersen 1940). The situation has taken on increased importance with the post 1990 computational boom in on-line data capture and analysis that has resulted in the measurement and evaluation of whole concentration distributions in an ultracentrifuge cell with time rather than the pre-1990 focus on the movement of sedimenting boundary positions with time.

The earlier approaches of measurement of weighted average sedimentation coefficients and their assigned true sedimenting concentrations are still valid—and indeed are applied—but these do not make full value of the data now available from single runs. Attempts to do so have thus far been thwarted by shortcomings in current instrumentation and the failure to detect the radial position of the air-liquid meniscus position with sufficient accuracy.

Nevertheless the lessons learnt — as well as the procedures established prior to the software boom — are still of value for the latter. This article reviews those developments and how they can be useful for the evaluation of whole concentration distribution analyses, and how a re-introduction of an optical system that has been discarded in modern instrumentation — the Schlieren optical system — can be useful in this regard, particularly as dilute solution treatments are being extended to the case of highly concentrated systems (>100 g/L) to cover the increasingly important case of high concentration protein therapeutics.

### The traditional “single weight averaged s” approach to radial dilution

The fact that centrifugal migration was being defined in terms of a single value of *s* led Kegeles and Gutter (1951) to conclude that the measured sedimentation coefficient should be regarded as an average parameter $\overline{s}$ that refers to the corresponding mean concentration $\overline{\mathrm{c}}$ (average of the plateau concentrations in the initial and final sedimentation velocity distributions used to delineate the magnitude of $\overline{s}$) — a practice that we would like to encourage for analyses, despite its relative antiquity.

## Early quantification of centrifugal migration

Sedimentation velocity experiments entail the use of a rotor speed that is sufficiently high to generate concentration distributions exhibiting a boundary between solvent and solution plateaux that migrates away from the air–liquid meniscus in response to the centrifugal field. Initially, this centrifugal migration of a macromolecular solute was quantified in terms of a sedimentation constant *s* (Svedberg and Rinde 1924), the rate of boundary movement divided by the force effecting that movement.

Specifically,

1 $$ s=\frac{dr/ dt}{\upomega^2r}=\frac{d\left(\ln r/ dt\right)}{\upomega^2} $$

where *dr*/*dt* describes the rate of boundary migration under the influence of the centrifugal field ω^2^*r* (product of radial distance *r* and the square of the angular velocity of rotation *ω*). A sedimentation constant was therefore determined from the slope (sω^2^) of the time dependence of ln *r*_*b*_, where *r*_*b*_ denotes the radial position of the boundary at time *t*. The inadequacy of this definition of centrifugal migration in terms of a single parameter was exposed soon after the availability of an electrically driven ultracentrifuge (the Spinco Model E) by the detection of a systematic variation of *s* with the concentration of protein subjected to velocity sedimentation — a situation that led to the reclassification of *s* as a sedimentation coefficient.

The problem of using the slope of an essentially linear time-dependence of ln *r*_*b*_ [Eq. (1)] to define a concentration-dependent parameter was taken into account initially (Kegeles and Gutter 1951) by regarding the parameter as an average sedimentation coefficient $\overline{s}$ over the range of plateau concentrations used for its measurement: the corresponding concentration $\overline{c}$ was therefore taken as the mean of those for the initial and final sedimentation distributions used for the measurement of $\overline{s}.$ Because the experimental sedimentation distributions were being monitored by the schlieren optical system (*dc*/*dr* vs *r*) in those days, the plateau concentration for a given distribution, *c*_*p*_, at time *t* could be calculated from the initial concentration, *c*_*o*_, as

2 $$ {c}_p={c}_o\exp {\left({r}_m/{r}_b\right)}^2={c}_o\exp \left(-2{\upomega}^2 st\right) $$

where *r*_*m*_ denotes the radial position of the air-liquid meniscus; and where the second form of the dependence follows from Eq. (1).

From a theoretical viewpoint, the preferred course of action is to express concentration dependence of the sedimentation coefficient for a macromolecular solute in terms of the value of *s* in the limit of zero solute concentration (*s*^*o*^) and a concentration coefficient (*k*_*s*_) as, correct to first order in concentration *c*:

3 $$ \left(1/s\right)=\left(1/{s}^o\right)\left(1+{k}_{\boldsymbol{s}}c\right) $$

Equation (3) used in early investigations to estimate *s*^o^ and *k*_*s*_ from the concentration dependence of 1/*s* for porous polymers (Kraemer and Lansing 1933; Signer and Gross 1934), polysaccharides (Gralén 1944)—the source of the name “Gralen coefficient” for *k*_s_, and highly asymmetrical but rigid macromolecules such as DNA (Cecil and Ogston 1948) —systems exhibiting a relatively large *s*–*c* dependence. For compact and more symmetrical macromolecular solutes such as globular proteins a smaller extent of concentration dependence gives rise to an essentially linear *s–c* dependence that can be described with adequate precision by an approximate form of Eq. Eq. (3) namely

4 $$ s={s}^o\left(1-{k}_sc\right) $$

Although the following considerations are presented in the context of quantifying the sedimentation behaviour of systems in terms of this simplified relationship, they are also relevant to systems for which Eq. (4) is required to describe the *s*–*c* dependence.

## Theoretical approaches to concentration assignment

The need to make allowance for a time-dependent sedimentation coefficient because of the continually decreasing solute concentration ahead of the migrating boundary was emphasized in studies of tobacco mosaic virus (Lauffer 1944), for which the extent of the concentration dependence sufficed to generate a nonlinear time-dependence of ln *r*_*b*_. That situation prompted the practice of calculating a time-dependent sedimentation coefficient, *s*^∗^(*t*), via the integrated form of Eq. (1), namely

5 $$ {s}^{\ast }(t)=\frac{\ln\ \left({r}_b/{r}_m\right)}{\upomega^2t} $$

where *t* is the time of centrifugation at angular velocity *ω* for boundary migration from the meniscus to *r*_*b*_. In the absence of that absolute timescale, an effective time needed to be estimated by plotting ln *r*_*b*_ versus (*t* − *t*_1_), the time expired since the first recorded distribution at angular velocity *ω*, and back-extrapolating to ln *r*_*m*_ in order to obtain an effective time *t*_1_ (at angular velocity *ω*) for that first distribution, and hence of the effective time of centrifugation (*t*) for subsequent distributions.

Three different theoretical approaches have all yielded the same quantitative expression, namely

6 $$ {s}^{\ast }(t)={s}^o\left(1-{k}_s{c}_o\right)+{s}^o\left(1-{k}_s{c}_o\right){k}_s{c}_o{\upomega}^2{s}^ot+\dots $$

for variation of the time-dependent sedimentation coefficient upon initial concentration loaded into the ultracentrifuge cell (*c*_*o*_) for systems exhibiting sedimentation velocity behaviour consistent with Eq. (4). Whereas Trautman et al. (1954) considered centrifugal migration for a diffusion-free system, Alberty (1954) deduced the same expression on the basis of the progressive decrease in plateau concentration effected by radial dilution [Eq. (2)] and the consequent variation in *s*^∗^(*t*). Subsequently, Fujita (1956) employed his approximate solution of the Lamm equation to incorporate the consequences of diffusional spreading on concentration distributions reflecting linear *s*–*c* dependence; and thereby to verify the validity of Eq. (6) by means of the time dependence of the boundary midpoint (*r*_*b*_) and hence of *s*^∗^(*t*) for a given *c*_*o*_.

From a theoretical viewpoint, it is evident that the application of Eq. (6) to sedimentation velocity distributions from an experiment with loading concentration *c*_*o*_ has potential for determination of the sedimentation coefficient *s*^†^ = *s*^o^(1 − *k*_*s*_*c*_*o*_) as the ordinate intercept of the time dependence of *s*^∗^(*t*), whereupon the magnitude of the concentration coefficient (*k*_*s*_) would follow from the dependence of *s*^†^ upon *c*_*o*_ in a series of experiments conducted with a range of initial protein concentrations. Indeed, Fujita (1956) has commented on the possibility of choosing a sufficiently small time of centrifugation (*ω*^2^*s*^0^*t* ≈ 0) to justify neglect of the contribution of the second term of the right-hand side of Eq. (6) to *s*^†^, whereupon the measured sedimentation coefficient would become the value associated with the loading concentration *c*_*o*_. Such theoretical logic does, of course, presume the availability of experimental sedimentation coefficients with the level of precision required for advantage to be taken of the above theory.

## Experimental considerations

The purpose of this section is to draw attention to the experimental results that evoked development of the above theoretical approach to determining the sedimentation coefficient *s*^†^ pertinent to the loading concentration *c*_*o*_; and then to expose the experimental limitations of sedimentation coefficient measurement that preclude its application as a reliable means of quantifying linear *s*–*c* dependence exhibited by globular proteins. To facilitate those considerations we first illustrate the features of theoretically predicted concentration distributions upon which the analyses are based. For that purpose advantage is taken of Eq. (6) to calculate the time-dependence of *s*^∗^(*t*) and hence ln (*r*_*b*_/*r*_*m*_), via Eq. (5), for a system with defined *s*–*c* dependence. Specifically, knowledge of *s*^o^ and *k*_*s*_ allows evaluation of the theoretical time-dependence of *s*^∗^(*t*) for assigned values of angular velocity (*ω*) and loading concentration (*c*_*o*_); and hence of ln *r*_*b*_ by designating the position of the boundary at zero time – the air–liquid meniscus (*r*_*m*_). A value of the plateau concentration pertinent to a given boundary position can then be calculated from Eq. (2).

**Fig. 1:**
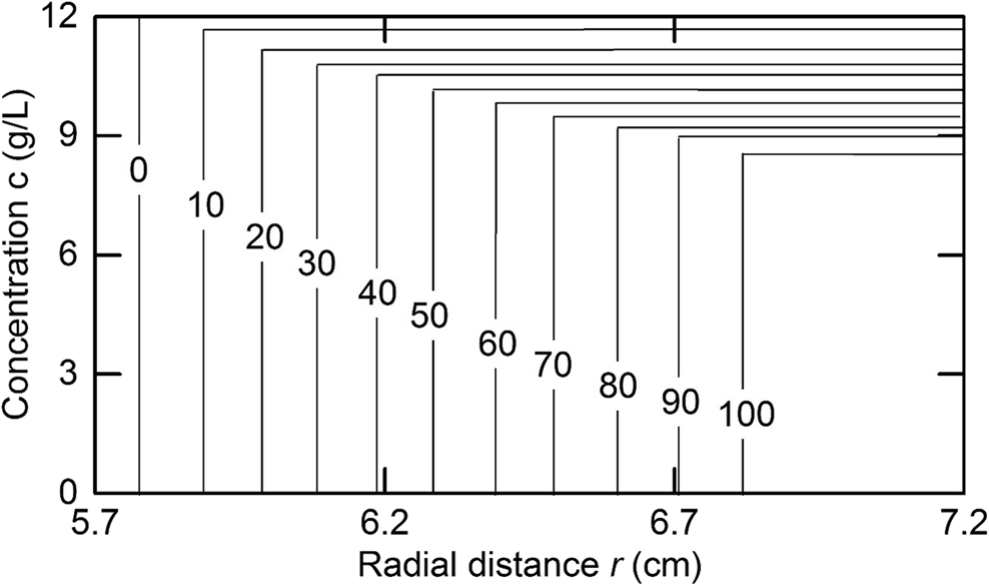
Effect of radial dilution on the plateau concentration in simulated asymptotic (diffusion-free) sedimentation velocity distributions for a 12-g/L solution of equine γ-globulin (*s*^o^ = 7.38 S, *k* = 0.00785 L/g) subjected to centrifugation at 60,000 rpm for the indicated times (min)

This approach to generating asymptotic (diffusion-free) concentration distributions is illustrated for a protein with the sedimentation velocity characteristics of equine γ-globulin, *s*^o^ = 7.38 S, *k*_*s*_ = 0.00785 L/g (Creeth 1964), a system for which the magnitude of the concentration coefficient is typical of that for globular proteins (0.007–0.008 L/g). The time-dependence of concentration distributions calculated by this means for a 12 g/L solution subjected to centrifugation at 60,000 rpm for 100 minutes is presented in Fig. 1, which highlights the progressive decline in plateau concentration *c*_*p*_ as the result of radial dilution—a phenomenon neglected in traditional measurements of sedimentation coefficients and brought to light in early studies of tobacco mosaic virus.

### Sedimentation velocity studies of tobacco mosaic virus

**Fig. 2:**
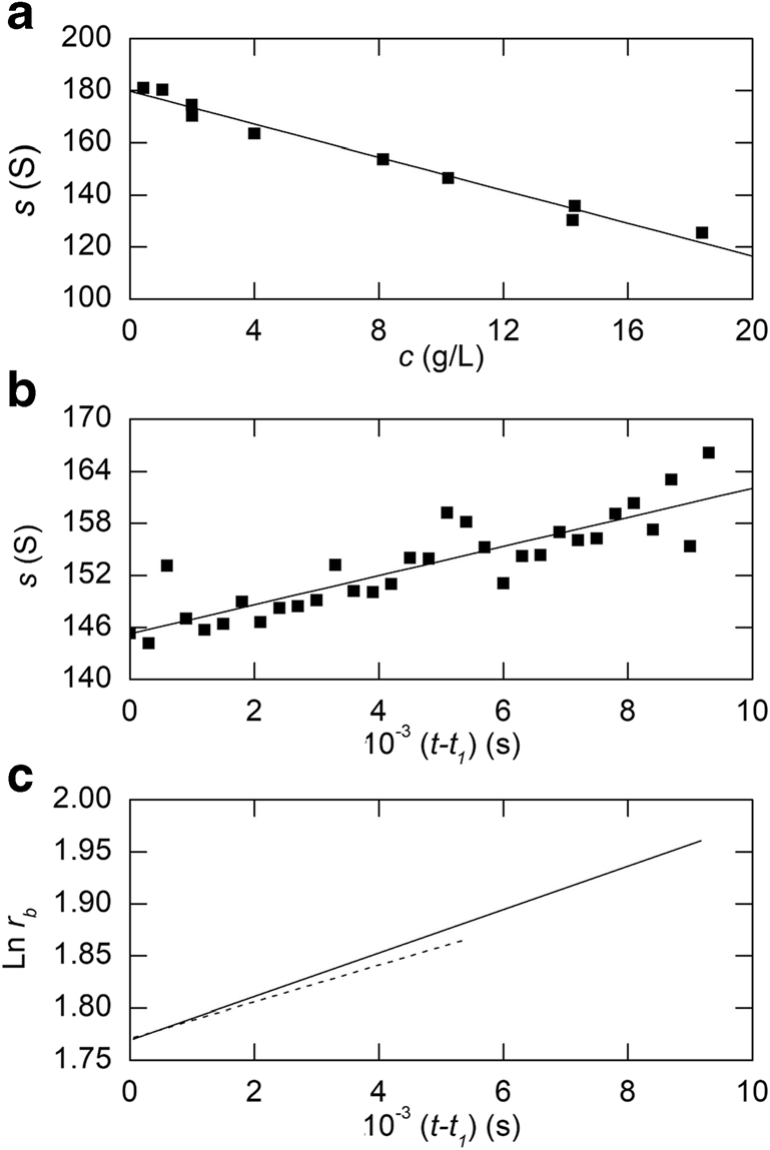
Early sedimentation velocity studies of tobacco mosaic virus. (**a**) Graphical representation of the concentration dependence of the sedimentation coefficient reported in Table I of Lauffer (1944). (**b**) Time-dependence of the measured sedimentation coefficient as the result of radial dilution, the data being taken from Table II of Lauffer (1944). (**c**) Corresponding time-dependence of the logarithm of boundary position that is predicted by the best-fit linear description of the data in (**b**): the limiting tangent (broken line) is included to highlight the curvilinearity of the predicted dependence

Analysis of the results reported in Table I of Lauffer (1944) for tobacco mosaic virus in 0.1 M phosphate buffer (pH 7.0) in terms of Eq. (3) signified values of 0.0054 S^–1^ for 1/*s*^o^ (or 185S for *s*^o^) and 0.0278 L/g for *k*_s_ (Lauffer 1944). However, those results are also described adequately in terms of linear *s*–*c* dependence (Fig. 2a), which necessarily yields a similar value for *s*^0^ (180 S) but a smaller magnitude for the linear concentration coefficient (*k*_*s*_= 0.0174 L/g). This disparity between concentration coefficients deduced from Eq. (3) and Eq. (4) reflects the limitations of (1 – *k*_*s*_*c*) as the expanded form of 1/(1 + *k*_*s*_*c*). Indeed, the fact that the concentration range covered in Fig. 2a incorporates  *k*_s_*c *values approaching 0.5 means that the binomial expansion needs to be extended to at least the sixth power in *k*_s_*c *for convergence of the series and agreement between the magnitudes of the concentration coefficients that are determined.

Of greater importance in the current context is the justification afforded by Fig. 2a for including tobacco mosaic virus as a system for which linear *s*–*c* dependence provides a reasonable (albeit operational) description of its sedimentation behaviour.

The extent of concentration dependence of the sedimentation coefficient for tobacco mosaic virus sufficed to demonstrate the progressive increase in *s* with distance migrated (*r*_*b*_) as the result of radial dilution of the solute plateau region ahead of the boundary [Table II of Lauffer 1944]. Those reported sedimentation coefficients, deduced from the difference in ln *r*_*b*_ over successive 300-second intervals of centrifugation of a 23.35 g/L solution at 11,100 rpm (*T* = 25.5 °C), are plotted as a time dependence in Fig. 2b, where the line denotes the best-fit linear description, *s* = [144 + 0.0016(*t* –*t*_1_)] S, that was inferred therefrom by Alberty (1954). Expression of the time dependence with that (*t*_1_) for the first recorded distribution as origin reflected the absence of a value for the position of the air–liquid meniscus (*r*_*m*_). In order to obtain the time-dependence of ln *r*_*b*_ for such a system, Eq. (5) with (*t* –*t*_1_) substituted for *t* and *r*_1_for *r*_*m*_, has then been used to infer *r*_*b*_ estimates from the best-fit *s* values, and hence to demonstrate curvilinearity of the consequent time-dependence of ln *r*_*b*_ (Fig. 2c, solid line).

**Fig. 3:**
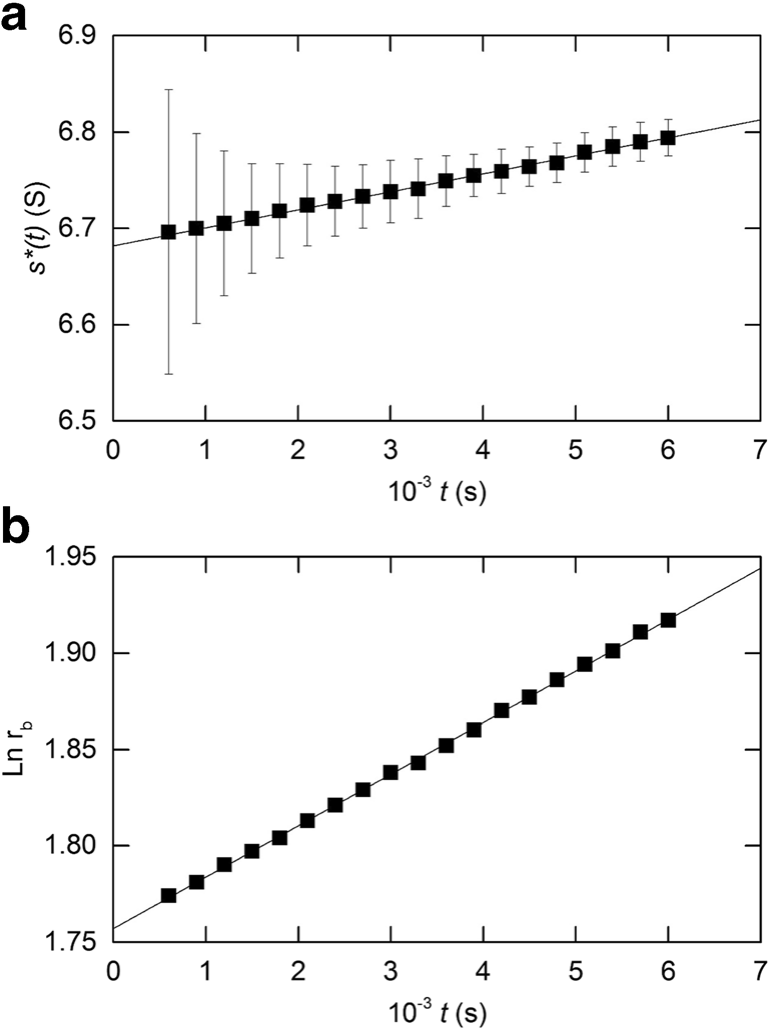
Calculated sedimentation velocity behaviour of a protein with the ultracentrifugal characteristics of equine γ-globulin (*s*^o^ = 7.38 S, *k* = 0.00785 L/g). (a) Time-dependence of the sedimentation coefficient *s*(*t*) predicted by Eq. (5) from the corresponding boundary positions in Fig. 1. (b) Corresponding time-dependence of the logarithm of the boundary position, which is essentially linear despite the progressive increase in *s*^∗^(*t*)

Although those early results for tobacco mosaic virus lack the precision required for accurate quantification of the *s*–*c* dependence, they clearly served not only to highlight the need to allow for the consequences of radial dilution, but also to influence the manner in which that allowance was incorporated into the theoretical expressions for the analysis of sedimentation velocity distributions reflecting concentration dependence of the sedimentation coefficient (Trautman et al. 1954; Alberty 1954; Fujita 1956).

## Predicted behaviour for globular proteins

We begin this section on the quantification of *s*–*c* dependence for compact globular protein systems by returning to the analysis of the calculated distributions (Fig. 1) for equine γ-globulin. In that regard, it should be noted that the extent of the progressive decline in plateau concentration (*c*_*p*_) is essentially the same for all macromolecular solutes because the duration and speed of centrifugation are selected to achieve similar extents of boundary migration. However, the consequences of that concentration decrease on the corresponding increase in the time-dependent sedimentation coefficient *s*^∗^(*t*) decrease markedly with molecular size because of their dependence on *k*_*s*_*cs*^o^. For example, although the concentration coefficient of 0.0174 L/g deduced from Fig. 2a for tobacco mosaic virus is little more than twice that (0.00785 L/g) for equine γ-globulin, the difference between the two concentration dependencies becomes huge when expressed in absolute terms: *s* = (180 – 3.11*c*) S for the virus compared with *s* = (7.38 – 0.058*c*) S for the globulin. That the problem of quantifying experimentally the change in *s*^∗^(*t*) with plateau concentration *c*_*p*_ in a single sedimentation velocity experiment becomes progressively greater with decreasing size of the globular protein is exemplified by the situation for ovalbumin, for which the values of 3.42 S for *s*^o^ and 0.0076 L/g for *k*_*s*_ translate into an absolute concentration dependence of *s* = (3.42 – 0.026*c*) S (Creeth and Winzor 1962). The question at issue therefore becomes the practical feasibility of taking advantage of Eq. (6) to obtain *s*^†^, the sedimentation coefficient for the solution with initial concentration *c*_*o*_, as the ordinate intercept of the time dependence of *s*^∗^(*t*).

As required, the application of Eq. (6) to the concentration distributions presented in Fig. 1 generates a linear time-dependent increase in the calculated values of *s*^∗^(*t*) as well as an ordinate intercept (6.685 S) that matches the sedimentation coefficient for the initial concentration *c*_*o*_, 12 g/L (Fig. 3a). However, the size of the vertical bars which indicate the effect of incorporating an uncertainty of 0.001 cm into the measurements of *r*_*b*_ and *r*_*m*_ (Baldwin 1957) on an experimental estimate of *s*^∗^(*t*) signifies the unlikelihood of reliable estimation of *s*^†^from the ordinate intercept.

Further evidence of the potential inability to detect let alone quantify experimentally the systematic but slight variation in *s*^∗^(*t*) is provided by the time-dependence of ln *r*_*b*_ reported for the γ-globulin in Fig. 3b, where the extent of curvilinearity is sufficiently small to justify the determination of an average sedimentation coefficient ($\overline{s}$) by linear regression analysis — the stance taken by Kegeles and Gutter (1951). Such action is verified by the return of a reasonably precise estimate (± 2SD) of (6.806 ± 0.013) S from the slope — a value that clearly differs from that (*s*^†^) of 6.685 S for the initial (12 g/L) solution. In that regard, it should be added that the existence of curvature in this error-free data is manifested in the associated value of (5.797 ± 0.001) for the ordinate intercept, which underestimates slightly the input meniscus position (*r*_*m*_) of 5.800 cm.

The same situation also applied to the corresponding analysis of calculated asymptotic concentration distributions for a 12 g/L solution of ovalbumin in that the ordinate intercept of 3.108 S for the time-dependence of *s*^∗^(*t*) again reproduced the value of *s*^†^for this system with *s*^o^ = 3.42 S and *k*_*s*_= 0.0076 L/g (Creeth and Winzor 1962). Likewise, the essentially linear time-dependence of ln *r*_*b*_ yielded an average sedimentation coefficient ($\overline{s}$) of (3.154±0.011) S which overestimated that (*s*^†^) of 3.108 S for a 12 g/L ovalbumin solution, as well as the slight underestimate of 5.797 (± 0.001) cm for the meniscus position (*r*_*m*_) of 5.800 cm.

The conclusion drawn from Fig. 3a that the ever-increasing uncertainty in *s*^∗^(*t*) with decreasing time of centrifugation precludes the evaluation of *s*^†^ as the ordinate intercept of the time dependence of *s*^∗^(*t*) was recognized by Baldwin (1957), who proposed an alternative method of allowance for the effect of radial dilution in a sedimentation velocity study of bovine serum albumin.

### The Baldwin approach

Having realized the shortcomings of the above approach, Baldwin (1957) rearranged Eq. (6) to the form

7 $$ {s}^{\ast }(t)={s}^o\left[1-{k}_s\left(1-{\upomega}^2{s}^ot\right){c}_o-{k}_s^2{\upomega}^2{s}^ot{c}_o^2\right] $$

which emphasizes the fact that for a fixed effective time of centrifugation *t*_*f*_ at angular velocity *ω*, *c*_*o*_ becomes the only variable parameter in the right hand side of Eq. (7). Extrapolation of the predicted linear dependence of *s*^∗^(*t*_*f*_) upon *c*_*o*_ to the ordinate intercept was therefore used to obtain an estimate of *s*^o^, wherepon advantage was taken of the following linear transform of Eq. (7),

8 $$ \frac{s^o-{s}^{\ast}\left({t}_f\right)}{s^o{c}_o}={k}_s\left(1-{\upomega}^2{s}^o{t}_f\right)+{k}_s^2{\upomega}^2{s}^o{t}_f{c}_o $$

to obtain estimates of the concentration coefficient *k*_*s*_ from the ordinate intercept, *k*_*s*_(1 − ω^2^*s*^o^*t*_*f*_) as well as the slope, ${k}_s^2{\upomega}^2{s}^o{t}_f$, of the dependence of [*s*^*o*^− *s*^∗^(*t*_*f*_)]/(*s*^o^*c*_*o*_) upon the initial loading concentration, *c*_*o*_.

**Fig. 4:**
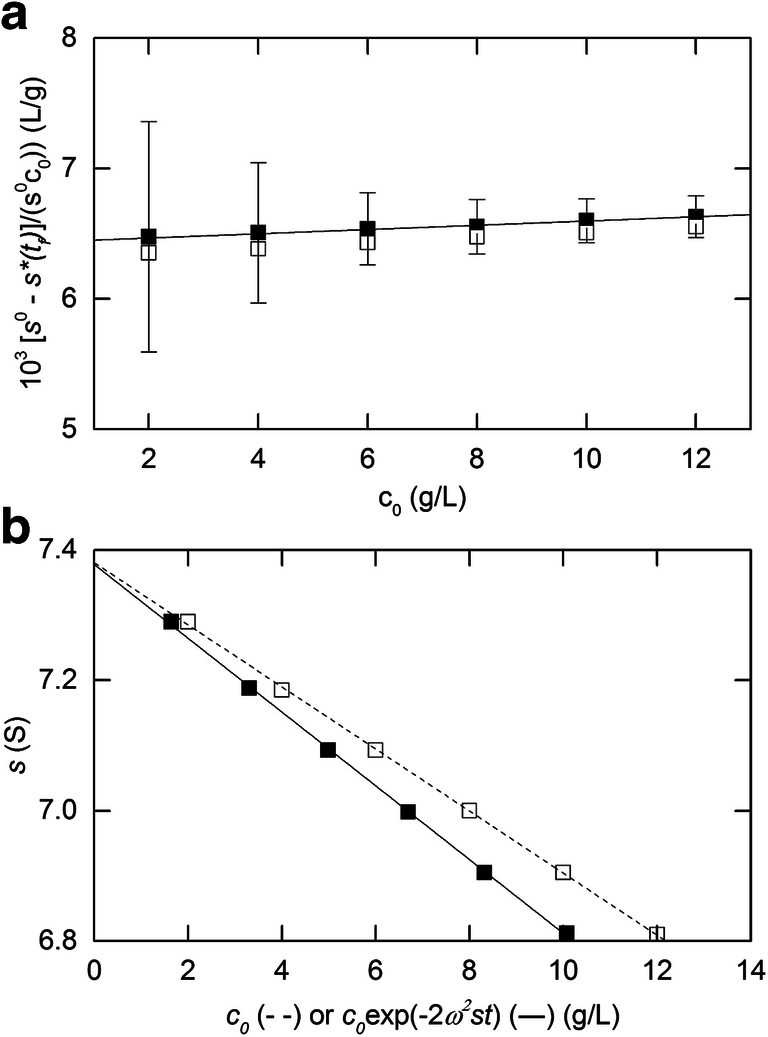
Application of the Baldwin approach to quantifying *s*^o^ and *k*_*s*_ for a protein with the sedimentation velocity characteristics of equine γ-globulin. (a) Analysis of the concentration dependence of *s*(*t*) data (■) according to Eq. (8) with the fixed time of centrifugation (*t*_*f*_) set at 6000 s, as well as the corresponding analysis (□) with the mean sedimentation coefficient ($\overline{s}$) substituted for *s*^∗^(*t*_*f*_). Error bars signify the consequences of an uncertainty of 0.001 cm in *r*_*m*_ and *r*_*b*_. (b) Dependence of the mean sedimentation coefficient $\overline{s}$ upon concentration defined in terms of loading concentration *c*_*o*_ (□) and the plateau concentration at time $\overline{t}$, the mean of those for first and last sedimentation velocity distributions used to delineate $\overline{s}\ \left(\blacksquare \right)$

That approach is illustrated in Fig. 4a, where the solid symbols denote analysis of the error-free data (rounded to the fourth significant figure) for equine γ-globulin for *t*_*f*_ = 6000 s in accordance with Eq. (8): the vertical lines again reflect the effect of a 0.001cm uncertainty in the measurements of *r*_*b*_ and *r*_*m*_ (Baldwin 1957). The first point to note is that the uncertainty problem inherent in the extrapolation of *s*^∗^(*t*) values to obtain *s*^†^ from a single experiment (the earlier approach illustrated in Fig. 3a) is not eliminated by employing a fixed time of centrifugation to analyze distributions from sedimentation velocity experiments with a range of loading concentrations *c*_*o*_. Instead, the change in approach has introduced a similar problem because of the increased uncertainty in the ordinate parameter, [*s*^o^ − *s*^∗^(*t*_*f*_)]/(*s*^o^*c*_*o*_), with decreasing loading concentration *c*_*o*_ — a factor that clearly mitigates against reliable estimation of *k*_*s*_ from either the ordinate intercept or the slope of the linear concentration dependence.

A second point to note from Fig. 4a is the relatively small contribution of the concentration-dependent term in Eq. (8) to the magnitude of the dependent variable,

[*s*^o^ − *s*^∗^(*t*_*f*_)/(*s*^*o*^*c*_*o*_)]. Although identical and correct values of *k*_*s*_ (0.00785 L/g) necessarily emanate from the returned magnitudes of the ordinate intercepts and slopes of the error-free data analysed in Fig. 4a, a far less satisfactory outcome would result from the superimposition of random error on the estimates of *s*^∗^(*t*_*f*_). To that, end we note the essentially linear time-dependence of ln *r*_*b*_ for these exact data (Fig. 3b) and hence the likelihood of indistinguishable *s*^∗^(*t*_*f*_) and $\overline{s}$ values. This possibility is also explored in Fig. 4a, where the open symbols reflect substitution of the mean sedimentation coefficient for each initial concentration for *s*^∗^(*t*_*f*_) in the analysis according to Eq. (8). Because this second set of values is within the experimental uncertainty envelope of those based on *s*^∗^(*t*_*f*_), the procedure suggested by Baldwin (1957) yields a sedimentation coefficient that is essentially $\overline{s}$; and should not therefore be identified with the loading concentration *c*_*o*_. These observations clearly expose an experimental limitation of the suggested approach (Baldwin 1957) for quantifying the concentration coefficient from the consequences of radial dilution for systems with the relatively small *s–c* dependence exhibited by globular proteins. Indeed, the rather low value of 0.0060 L/g is obtained for *k*_*s*_ by this procedure for bovine serum albumin (Baldwin 1957), and we provide further comment on this later.

The practice (Kegeles and Gutter 1951) of regarding the measured sedimentation coefficient as an average parameter ($\overline{s}$) related to the corresponding mean plateau concentration $\overline{c}$ at time $\overline{t}$ (the average of *t* for the first and last distributions used for evaluating $\overline{s}$) thus remains a preferred procedure for quantifying the *s*–*c* dependence for globular proteins. This is evident from Fig. 4b, which presents the dependence of $\overline{s}$ upon initial concentration *c*_*o*_ (open symbols) and the plateau concentration,$\overline{c}={c}_o\exp \Big(-2{\upomega}^2\overline{s}\overline{t}$) (closed symbols), for the above error-free data for a protein with the sedimentation velocity characteristics of equine γ-globulin.

### Summary of early approaches to experimentally quantify *s*-*c* dependence

Despite their theoretical feasibility, attempts to quantify *s–c* dependence in terms of the loading concentration *c*_*o*_ by means of time-dependent sedimentation coefficients *s*^∗^(*t*) as the result of radial dilution in a *single* sedimentation velocity experiment (Trautman et al. 1954; Alberty 1954; Fujita 1956) are thwarted by experimental limitations encountered with globular proteins. Unfortunately, the decrease in protein concentration effected by radial dilution is too small for the extent of the predicted time-dependent increase in *s*^∗^(*t*) to exceed the experimental uncertainty inherent in the measurement of the sedimentation coefficient (Fig. 3a). That conclusion has been based on an experimental uncertainty of 0.001 cm in the measured positions of the boundary (*r*_*b*_) and air–liquid meniscus (*r*_*m*_) — a value considered by Baldwin (1957) to apply to concentration distributions recorded by the schlieren optical system (d*c*/d*r* vs *r*) of the Spinco (later Beckman) model E analytical ultracentrifuge at that time. Consequently, the estimates of *s*^∗^(*t*) became essentially indistinguishable from $\overline{s}$, the average value obtained via Eq. (1) by assuming constancy of the sedimentation coefficient over the time period of the *s*^∗^(*t*) measurements. A similar uncertainty problem has also led to the downfall of the subsequent procedure (Baldwin 1957) in which a fixed time of centrifugation (*t*_*f*_) is used to assess the dependence of *s*^∗^(*t*_*f*_) upon loading concentration (Fig. 4a). This effectively left the Kegeles-Gutter approach as the only appropriate method.

Admittedly, the above considerations refer specifically to methods of sedimentation coefficient measurement that are now regarded as archaic because of their development in an era when analytical integration of the differential equation describing centrifugal migration was a prerequisite for its application to experimental sedimentation velocity distributions. In that regard the situation was certainly not helped by the virtual demise of analytical ultracentrifugation soon after their development. Indeed, two decades elapsed before interest in the technique was revitalized by the appearance of a new-generation instrument in the final decade of the 20th century. The advent of the Beckman XL-A and XL-I centrifuges with online data capture in the form of absorbance and Rayleigh interference distributions coincided with the advances in computer technology that rendered nonlinear differential equations readily amenable to solution by numerical quadrature and integration - an advance that has revolutionized the way in which sedimentation velocity experiments are analyzed and interpreted. Furthermore, the need for estimating an effective time of centrifugation has been eliminated by the provision of a continuous record of that parameter $\left({\int}_0^t{\omega}^2 dt\right)$ from the commencement of rotor rotation.

## Measurement of sedimentation coefficients in the on-line computer age

By the 1990s, computer technology had advanced to the extent that consideration could now be given to the development of procedures for the determination of sedimentation coefficients by the analysis of sedimentation velocity distributions in terms of the complete differential equation for solute migration in a centrifugal field (Lamm 1929), namely

9 $$ {\left(\frac{\partial c}{\partial t}\right)}_r=\frac{1}{r}\left[\left(\frac{\partial }{\partial r}\right){\left( rD{\left(\frac{\partial c}{\partial r}\right)}_t-{\omega}^2{r}^2 sc\right)}_t\right] $$

which includes the translational diffusion coefficient (*D*) to encompass boundary spreading. Analysis of experimental sedimentation velocity distributions by this means clearly requires attention to be given to the consequences of migration arising from this additional factor. As a further complication, *D* will also be dependent on concentration. For dilute solutions, an equation analagous to Eq. (4) is used:

10 $$ D={D}^0\left(1+{k}_dc\right) $$

### The g*(s) procedure

The initial approach to this problem entailed the generation of an apparent differential sedimentation coefficient distribution, *g*^∗^(*s*) vs *s*, for a hypothetical set of non-diffusing particles (Stafford 1992, 1994; Philo 1997, 2000, 2006; Stafford and Sherwood 2004; Sherwood and Stafford 2016). To generate that differential concentration distribution, defined by the expression

11 $$ {g}^{\ast }{(s)}_t=\left(\frac{\partial c}{\partial t}\right)\left(\frac{1}{c_o}\right)\left(\frac{r^2}{r_m^2}\right)\left(\frac{\upomega^2{t}^2}{\ln \left({r}_m/r\right)}\right) $$

from the experimental profile (*c* vs *r*), Stafford made the approximation that the derivative of concentration with respect to time could be replaced by Δ*c*/Δ*t*, the concentration difference Δ*c* at radial distance *r* in distributions recorded an incremental time difference Δ*t* apart. As no account is being taken of diffusional spreading, *g*^∗^(*s*) describes the apparent weight-fraction of material with sedimentations coefficients between *s* and (*s* + Δ*s*). Time-dependence of *g*^∗^(*s*) is removed by employing Eq. (5) with *r* substituted for *r*_*b*_ to generate a distribution with sedimentation coefficient *s* as the abscissa. Also, because no account has also been taken of the *consequences* of diffusional spreading on the form of the apparent distribution, the sedimentation coefficient is taken as the value of *s* corresponding to its median bisector (peak value for a symmetrical distribution). Philo (2000, 2006) has refined the analysis by using the resulting g*(*s*)-*s* distribution to calculate the corresponding best-fit description of the experimental distribution (Δ*c*/Δ*t* vs *s*)—a procedure available in the DCDT+ software package.

**Fig. 5:**
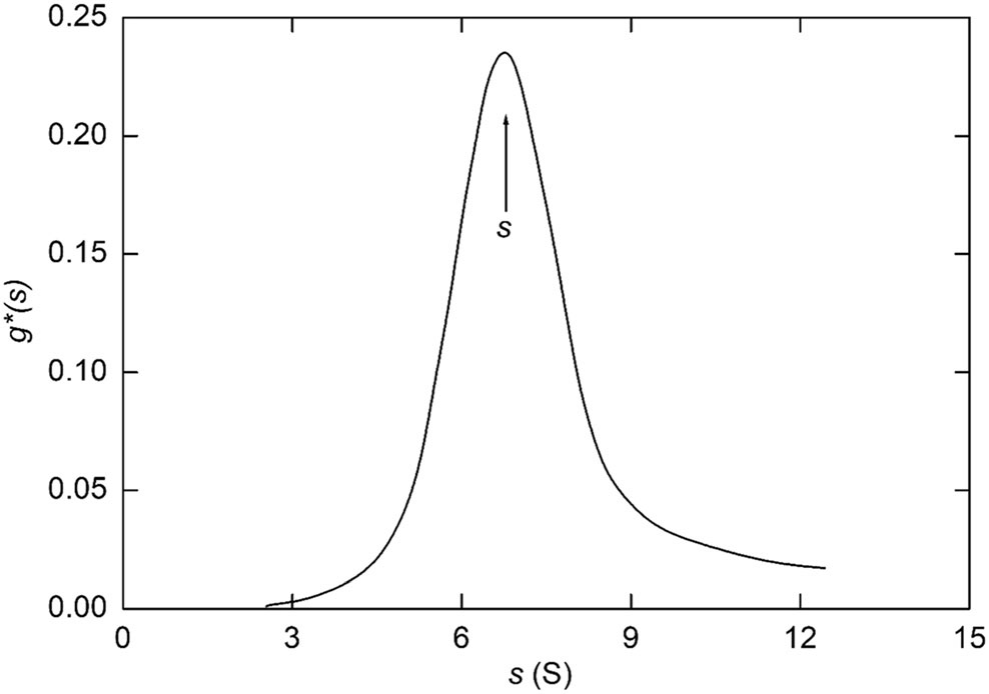
Illustration of the g*(*s*) procedure for determination of the sedimentation coefficient of a monoclonal antibody (IgG) to diphtheria toxin. [Data taken from Fig. 4 of Stafford 1992]

The *g*^∗^(*s*) procedure is illustrated in Fig. 5, which presents the distribution obtained (Stafford 1992) from Rayleigh interference patterns for a monoclonal antibody (IgG) to diphtheria toxin (*c*_*o*_ = 0.19 g/L) subjected to centrifugation at 56,000 rpm for 3384 sec. In that regard, the use of a low loading concentration has justified the approximation inherent in consideration of the system in terms of a fixed sedimentation coefficient, whereupon the returned value is essentially *s*^o^.

### The Van Holde-Weischet procedure

**Fig. 6:**
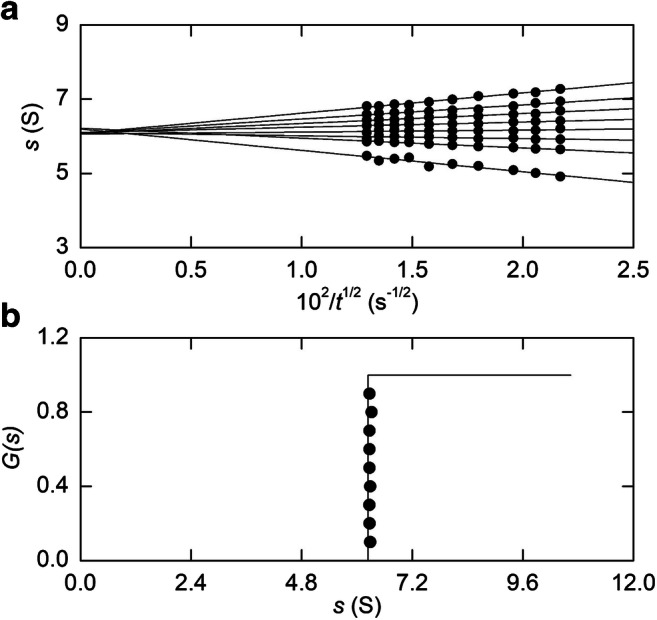
Evaluation of a sedimentation coefficient by the Van Holde-Weischet procedure. (a) Boundary analysis of sedimentation distributions (48,000 rpm) for fragment K of PM2 DNA in accordance with Eq. (11) to obtain *s* by the extrapolation of apparent sedimentations coefficients to infinite time. (b) Illustration of solute homogeneity by means of the asymptotic *G*(*s*) distribution derived therefrom. [Data in (a) has been taken from Fig. 4 of Van Holde and Weischet 1978]

A second means of overcoming the need of a value for *D* entails generation of the asymptotic (diffusion-free) pattern from the time-dependence of the sedimentation velocity distribution (Winzor et al. 1977; Van Holde and Weischet 1978). It exploits the fact that the contribution of diffusional spreading only varies with $\sqrt{t}$ whereas centrifugal migration exhibits a linear time dependence, whereupon the consequences of diffusional spreading can be eliminated by the extrapolation of *s*^∗^ values [Eq. (5)] to infinite time. In this procedure (Van Holde and Weischet 1978; Demeler et al. 1997; Demeler and Van Holde 2004) the ordinate of each distribution in an experiment conducted at angular velocity *ω* is divided into 10 equal increments Δ*c*, and the radial positions corresponding to *c*/*c*_*p*_= 0.05, 0.10, ….., 0.95) converted to sedimentation coefficients *s*^∗^ via Eq. (5). On the grounds that the time-dependence of s* is given by

12 $$ {s}^{\ast }=s-\left(\frac{2\sqrt{D}}{r_m{\upomega}^2}\right)\left[{\mathit{\operatorname{erf}}}^{-1}\Big(1-2\upomega \right]\left(\frac{1}{\sqrt{t}}\right) $$

where *erf*^−1^ denotes the inverse error function, the sedimentation coefficient *s* is obtained as the ordinate intercept of the dependence of *s*^∗^ upon $1/\sqrt{t}$ . This aspect of the analysis is illustrated in Fig. 6a for restriction enzyme fragment K of PM2 DNA (Van Holde and Weischet 1978). Introduction of an integral sedimentation coefficient distribution function *G*(*s*) as (∑Δc)/*c*_*p*_ then allows construction of a time-normalized asymptotic (diffusion-free) migration profile (Fig. 6b) — a counterpart of the time-dependent patterns presented in Fig. 2. As in Fig. 5, the use of a low loading concentration *c*_*o*_ (below 0.025 g/L) to eliminate effects of *s*-*c* dependence has ensured the return of *s*^o^ as the measured sedimentation coefficient (6.16 S).

Because the use of Eq. (5) to obtain *s*^∗^ values is predicated upon accurate location of the air-liquid meniscus position *r*_*m*_ in both procedures, their application is most relevant to the analysis of experimental sedimentation velocity distributions recorded by the absorption optical system of current analytical ultracentrifuges. Indeed, the relatively poor resolution of the air-liquid meniscus region in Rayleigh interference records of concentration distributions from the XL-I ultracentrifuge (Philo 1997; Schuck 2000; Brown et al. 2009) has led to the recommendation that *r*_*m*_ be best regarded as an additional curve-fitting parameter to emanate from the analysis of sedimentation velocity distributions in terms of Eq. (9) (Schuck 2000, 2005; Brown et al. 2009). The consequent problem in defining *s*^∗^ also extends to the third and most commonly used current procedure for sedimentation coefficient determination.

### The c(s) procedure

**Fig. 7:**
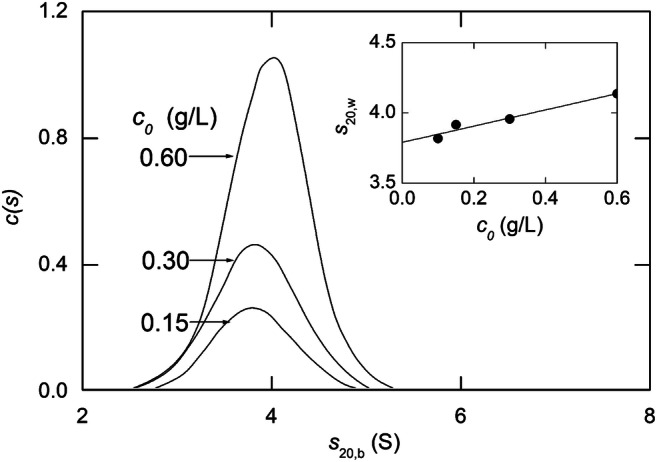
Evaluation of the sedimentation coefficient of a laminin short-arm fragment (0.6 g/L) in Tris-chloride buffer (pH 8.5, *I* 0.17) by *c*(*s*) analysis of absorbance distributions resulting from centrifugation at 35,000 rpm. [Data taken from Fig. 5C of Patel et al. (2016)]

Quantitative analysis of sedimentation velocity distributions for a single solute in terms of the Lamm equation [Eq. (9)] entails estimation of the translational diffusion coefficient (*D*) as well as the sedimentation coefficient (*s*) via a differential distribution function *c*(*s*) defined by the relationship (Schuck 1998, 2000)

13 $$ c\left(r,t\right)=\underset{s_{min}}{\overset{s_{max}}{\int }}{c}_s ds $$

where *s*_*max*_ and *s*_*min*_ are the extremes of sedimentation coefficient across the distribution (*s*^0^ and that for the plateau concentration in a distribution completely resolved from the air-liquid meniscus). Numerical integration of Eq. (9) is then used iteratively to obtain a best-fit description of a selected series of distributions with *s* and *D* as curve-fitting parameters. Indeed, because of uncertainty about the location of the air-liquid meniscus(*r*_*m*_), additional iteration is included to refine its magnitude as that associated with the smallest standard error in the estimate of *s*. The *c*(*s*)-*s* distribution for a laminin short arm fragment (Patel et al. 2016) is shown in Fig. 7, where the abscissa value associated with the peak defines the magnitude of the sedimentation coefficient. In this instance, the concentrations (0.15-0.60 g/L) would also be sufficiently small for identification of the sedimentation coefficient with the limiting value (*s*^0^) for a noninteracting species. However, the observed positive *s*−*c* dependence (inset to Fig. 7) necessitates consideration of the measured sedimentation coefficients as average values ($\overline{s}$) for a 3.8 S monomer undergoing reversible self-association (Patel et al. 2016). In retrospect, the slope of the line in the inset to Fig. 7 underestimates the actual extent of the *s*-*c* dependence because of incorrect substitution of *c*_*o*_ for $\overline{c}$ as the relevant concentration.

**Fig. 8:**
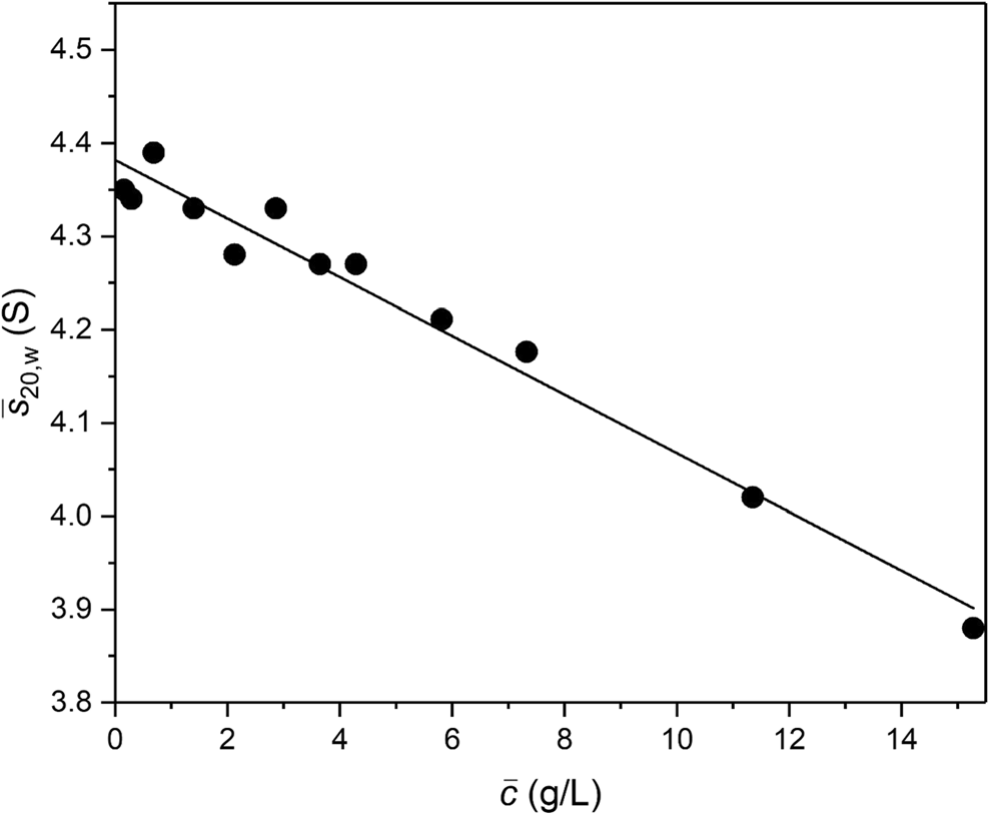
Quantification of the *s*-*c* dependence for bovine serum albumin in phosphate-buffered saline by plotting the sedimentation coefficient,${\overline{\ s}}_{20.w}$, obtained by the standard SEDFIT analysis of sedimentation velocity distributions as a function of the corresponding mean plateau concentration, $\overline{c}$ . Values of *s*^o^_20,w_ = (4.38± 0.02)S and *k*_s_ = (0.0072 ± 0.0003) L/g are returned

That possibility has been tested by experimental delineation of the *s*-*c* dependence with ($\overline{c}$, $\overline{s}$) as the information emanating from the time-dependence of sedimentation velocity distributions (Kegeles and Gutter 1951; Patel et al. 2018). Rayleigh interference records of concentration distributions from sedimentation velocity experiments (45,000 rpm and 20.0 °C) on 0-15.3 g/L solutions of bovine serum albumin (Sigma) in phosphate-buffered saline of ionic strength 0.1M (considered sufficient to suppress significant polyelectrolyte behaviour) were first converted to g*(s)-s distributions by the least squares g*(*s*) procedure within SEDFIT (Dam and Schuck 2004). Combination of the sedimentation coefficient associated with the peak g*(*s*) value ($\overline{s}$) with the mean plateau concentration ($\overline{c}$) used for its determination (Kegeles and Gutter 1951; Patel et al. 2018) yielded the *s*-*c* dependence shown in Fig. 8 (in the linear data region of 0-15 g/L) where the solid line denotes the best-fit description of the ($\overline{c}$, $\overline{s}$) data in terms of Eq. (4) and signifies values of (4.38 ± 0.02)S for *s*^o^ and (0.0072 ± 0.0003) L/g for *k*_*s*_. In that regard the lower estimate of (0.0059 ± 0.0002) L/g for *k*_*s*_ obtained by combining $\overline{s}$ with the loading concentration *c*_*o*_ mirrors the lower based on *s*^∗^(*t*_*f*_) that was reported by Baldwin (1957); and thereby verifies the earlier inference (Fig. 4) that the parameter designated as *s*^∗^(*t*_*f*_) is experimentally indistinguishable from $\overline{s}$.

### Allowance for radial dilution in the evaluation of sedimentation coefficients from a single experiment

Developments in finite element analysis have been incorporated into subsequent SEDFIT (Solovyova et al. 2001; Chaturvedi et al. 2018) and SEDANAL (Stafford and Sherwood 2004; Sherwood and Stafford 2016) programs to determine a point-by-point local concentration from which the limiting sedimentation coefficient (*s*^o^) and the concentration coefficient (*k*_*s*_) for a protein can be determined by nonlinear regression analysis. For example, the SEDFIT program incorporates concentration dependence of sedimentation and diffusion coefficients by means of the relationships

14a $$ s\left(r,t\right)={s}^0\left[1-{k}_sc\left(r,t\right)\right] $$

14b $$ D\left(r,t\right)={D}^0\left[1+{k}_Dc\left(r,t\right)\right] $$

An additional constraint on their magnitudes is imposed by expressing their interdependence as (Harding and Johnson 1985a, 1985b)

15 $$ 2{B}_2={k}_s+{k}_D $$

where *B*_2_ is the osmotic second virial coefficient expressed in mL/g [BM in the terminology of Harding and Johnson 1985a, 1985b—see also Tanford 1961 and Harding et al. 1999]. Equation 15 holds with the now standard practice of buoyancy corrections for the sedimentation coefficients calculated with respect to solvent rather than solution density—if the latter an additional term involving the partial specific volume $\overline{v}$ is needed (Harding and Johnson 1985a, 1985b).

**Fig. 9:**
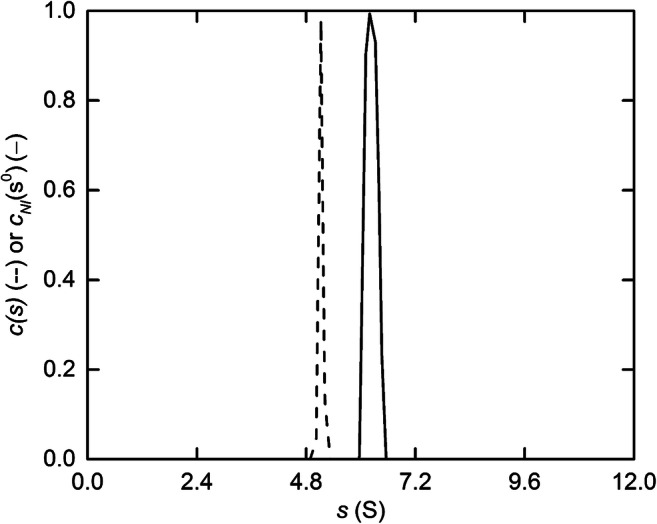
Allowance for concentration dependence of the sedimentation coefficient in the analysis of sedimentation velocity distributions for a reference monoclonal antibody (SRM 8671, NIST, Gaithersburg) centrifuged at 45,000 rpm in 25mM histidine buffer. The solid line describes the (monomer) distribution obtained by the refined *c*_NI_(*s*_o_) analysis, whereas the broken line is the corresponding distribution obtained by the standard *c*(*s*) procedure. A dimer peak compromising 2.7% of the material present is not shown. [Data taken with permission from Fig. 2 of Chaturvedi et al. 2018]

This approach, termed the *c*_NI_(*s*_o_) approach, is illustrated by the solid line in Fig. 9, which signifies a sedimentation coefficient (*s*^o^) of 6.56 S and a concentration coefficient (*k*_*s*_) of 0.0195 L/g (Chaturvedi et al. 2018) for a reference monoclonal antibody (IgG) preparation (10 g/L) in a standard low ionic strength solvent used for such preparations (25mM histidine) subjected to centrifugation at 45,000 rpm, and it means that *distributions* of sedimentation coefficient can now be corrected for non-ideality, rather than just average or peak/component sedimentation coefficients. The sedimentation coefficient estimate obtained by the standard *c*(*s*) analysis (- - -, Fig. 9) reflects the assumed constancy of *s* and hence its evaluation as $\overline{s}$ as discussed above in relation to Eq. (7) Another consequence of that assumption is the return of a sharpened distribution as the result of incorporating the boundary sharpening arising from *s*-*c* dependence into a smaller magnitude for the apparent diffusion coefficient. The large value of *k*_s_ is due to the low ionic strength of the standard solvent used with incomplete shielding of polyelectrolyte effects.

The inclusion of Eqs. (14a) and (14b) to allow for the changes in *s* and *D* effected by radial dilution in the *c*_NI_(*s*_o_) approach of SEDFIT has also been applied to solutions of BSA in PBS buffer (Chaturvedi et al. 2018) and a value for *k*_s_ of 0.0084 L/g is obtained. This is a little higher than the value found from conventional radial dilution methods (Fig. 8) of 0.0072 L/g, the difference possibly being due to the high initial loading concentration (52 g/L) used for the *c*_NI_(*s*_o_) analysis and its effects on the oligomeric state and applicability of the linear relations desctibed in Eqs. 4 and 14. This is discussed further below.

### Predicted extent of *D*-*c* dependence for globular proteins from sedimentation velocity experiments

Generally, diffusion coefficients obtained under the thermodynamic constraints of constant temperature and solvent chemical potential [those considered to operate in ultracentrifuge experiments (Braswell 1968, 1987; Wills et al. 1993, 2000; Winzor et al. 2004)] exhibit a smaller concentration dependence compared with sedimentation coefficients due to the opposing effects of the thermodynamic and hydrodynamic terms (Harding and Johnson 1985a).

Most consideration has been given to the quantitative description of *D*-*c* dependence under conditions where thermodynamic activity is being monitored under the constraints of constant temperature and solvent chemical potential that operate in the measurement of diffusion coefficients by the traditional boundary spreading technique (Gosting 1956) as well as in sedimentation velocity experiments (Braswell 1987; Winzor et al. 2004).

Theoretical considerations of the diffusion of globular protein modelled as a hard sphere with molar mass *M*, solvated radius *R* and net charge *Z* under those thermodynamic constraints (Petsev and Denkov 1992; Arzensěk et al. 2012; Harding and Johnson 1985a; Scott et al. 2014) give rise to the expression

16 $$ {k}_D=\left[8-{K}_s+f\left(Z,I\right)\right]{v}_s $$

for the concentration coefficient *k*_*D*_ (mL/g) expressed in terms of the hydrated specific volume *v*_*s*_ = 4π*N*_*A*_*R*^3^/(3*M*), and *K*_s_ = *k*_s_/*v*_*s*_. The polyelectrolyte term *f*(Z, I) = 0 for uncharged polymers and for proteins at the isoelectric pH. It will also be approximately zero for either charged polymers or for proteins not in isoelectric conditions so long as the ionic strength I is sufficiently high to shield the charges. The contribution of polyelectrolyte behaviour to the thermodynamic term, [*f*(*Z*, *I*)]^*EL*^, in Eq (16) has been well described [see, e.g. Petsev and Denkov (1992)] as

17 $$ {\left[f\left(Z,I\right)\right]}^{EL}=\left(\ \frac{Z^2}{2I\left(1+\upkappa R\right)M}\right) $$

where κ, the inverse screening length (Debye and Hűckel 1923) = 3.27 × 10^7^$\sqrt{I\ }{\mathrm{cm}}^{-1}$ at 20.0°C. Unfortunately, there is no corresponding expression yet available describing the polyelectrolyte contribution to *k*_s_ (or *K*_*s*_) and the assumption is made that solvent conditions are such that the charge contributions to both the thermodynamic and hydrodynamic terms are negligible or compensatory.

The correctness of *k*_*D*_ predicted by Eq. (16) also depends on the correctness of the sedimentation concentration term *K*_s_. Batchelor (1972) calculated that at sufficiently high dilution where pairwise hydrodynamic interactions between the particles only apply *K*_s_ = 6.55 for the hard-sphere approximation (a value later supported by other researchers—see. e.g. Felderhof (1988) and Cichocki and Felderhof (1990)). Use of that value of *K*_*s*_ in Eq. (16) yields the expression

18 $$ {k}_D=\left(1.45+f\Big(\mathrm{Z},\mathrm{I}\ \right)\Big){v}_s $$

In a detailed study (with sedimentation coefficients fully corrected for radial dilution using the Kegeles-Gutter method) on monodisperse solutions of the rigid spherical turnip yellow mosaic virus (TYMV) Harding and Johnson (1985b) showed that in a concentration range from 1 to 10 g/L (equivalent to a very low volume fraction ϕ = *c*.*v*_s_ of ~ 0.01) values for *K*_s_ ranged from 5.0 to 6.3 (for 4 different pH’s (4.75, 6.0, 6.8, 7.8) and 2 different ionic strengths (*I* = 0.1M and 0.2M) buffer. In terms of Batchelor theory these values refer to a solvent frame of reference as noted above. Under isoelectric conditions (pH 4.8), for which *Z* = 0 in Eq (16), *K*_s_=5.3. This suggested that, even allowing for experimental error and the effects of departure from exact sphericity of the TYMV virions, the pairwise approximation does not appear to give an exact representation except at very high dilution (*c* < 0.1 g/L, or volume fractions ϕ < 0.01), when the changes of *s* with *c* are no greater than the precision of the measurement.

Theoretical adjustments have been suggested by other researchers taking into consideration of multi-particle interactions or increased viscosity of the fluid due to the presence of neutrally buoyant particles (see for example Beenakker & Mazur 1983; Brady and Durlofsky 1988; Hayakawa and Ichiki 1995). Brady and Durlofsky’s multi-body approach suggested a value of about 5 for *K*_s_ . The lower value (which reduces to ~4 when the sedimentation coefficients are buoyancy corrected for solution rather than solvent density) fits better the experimental data for polystyrene latex spheres of Cheng and Schachman (1955) and a wide range of globular proteins examined by Creeth and Knight (1965) based on the Wales-van Holde ratio (*k*_s_/[η]): Cheng and Schachman (1955), and also Creeth and Knight (1965), found values for this ratio to be about 1.6 for globular particles. The lower value would also yield a value of approximately 3*v*_*s*_ mL/g for *k*_*D*_. Further adjustments have been indicated: for example, Scott et al. (2014) have suggested allowance for the viscosity of the solution yielding a value of approximately 0.5*v*_*s*_ mL/g for *k*_*D*_: if a similar correction is applied to the Batchelor-Felderhof value, *k*_*D*_ becomes - 1.05*v*_*s*_ mL/g.

**Fig. 10:**
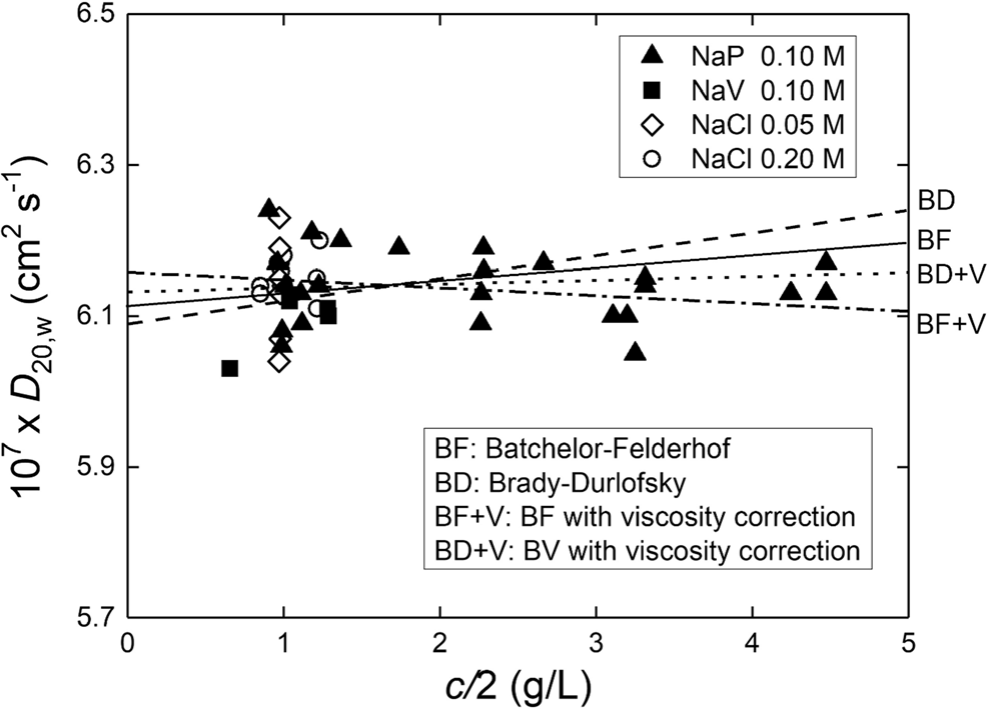
Concentration dependence of the translational diffusion coefficient for bovine serum albumin. Comparison of results [Table 4 of Creeth 1952)] obtained under various solvent conditions by the traditional boundary spreading procedure with theoretically predicted concentration dependences. The combined data are fitted to Batchelor-Felderhof and Brady-Durlofsky representations (with and without allowance for solution viscosity)

We can also see how the various theoretical values for *k*_*D*_ fit with experiment: Fig. 10 summarizes the concentration dependence of diffusion coefficients reported by Creeth (1952) for bovine serum albumin by the traditional procedure of monitoring the spreading of an initially sharp boundary between protein solution and diffusate with which it was in dialysis equilibrium. Because of the return of a single value of *D* from the spreading of a boundary between solvent and a solute solution with plateau concentration *c*, the measured diffusion coefficient must be regarded as an average value pertaining to the mean concentration *c*/2.

Those results, which refer mainly to albumin in phosphate buffer (pH 6.8, *I* 0.10M) but also include diffusion coefficients for albumin in 0.2 M NaCl as well as veronal buffer (pH 8.6, *I* 0.10M) signify relative insensitivity of *D* to protein concentration, pH and ionic strength of the medium. In the absence of any discernible concentration dependence (Fig. 10), the mean value of (6.14 ± 0.04) × 10^-7^ cm^2^s^-1^ for *D*_20, *w*_ has been used to calculate a Stokes radius of 3.5 nm and hence a specific solvated volume (*v*_*S*_) of ~ 1.6 mL/g. Using this value in Eq. (16), it is seen that after allowing for experimental error all four theoretical predictions considered (Batchelor-Felderhof, Brady-Durlofsky and with or without the suggested correction for solution viscosity) are consistent with the data, which are effectively constant across the range of concentrations considered (0-5 g/L for *c*/2). A higher value for *k*_*D*_ of 10 mL/g was later obtained by Chaturvedi et al. (2018) deduced as a curve-fitting parameter by incorporating Eq. (14b) into the analysis of boundary spreading for bovine serum albumin in a similar buffer. The loading concentration was much higher (52 g/L) than used in the Creeth experiments and as with the estimate for *k*_s_ this may reflect differences in the oligomeric state of the protein compared to the conditions used by Creeth in Fig. 10, i.e. the differences reflect different experimental conditions: this is the subject of further research.

It is worth also noting a different case for the measurement of diffusion coefficients under the thermodynamic constraints of constant temperature and pressure that pertain in dynamic light scattering experiments. Indeed, concentration dependence of the diffusion coefficient was incorporated into the boundary spreading analyses to accommodate positive *D*-*c* dependence such as that observed in dynamic light scattering studies of turnip-yellow-mosaic virus (Harding and Johnson 1985b). In principle, such results are not amenable to interpretation in terms of single-solute theory because of the need to regard small species such as buffer components and supporting electrolytes as additional non-scattering cosolutes (Kirkwood and Goldberg 1950; Stockmayer 1950; Hill 1959; Winzor et al. 2007); but the consequences of neglecting those protein-cosolute interactions turn out to be minor.

### Summary of current approaches to quantifying *s*-*c* dependence

As pointed out recently (Patel et al. 2018), the return of a single value for the sedimentation coefficient by the standard version of all of these computer procedures assumes constancy of *s*, and hence requires its identification as $\overline{s}$, to which a corresponding mean concentration $\overline{c}$ must be assigned (Kegeles and Gutter 1951). In the absence of any guidance on this point in the software packages, the usual but incorrect practice of substituting the loading concentration *c*_*o*_ for $\overline{c}$ leads to underestimation of the concentration coefficient *k*_*s*_ (Patel et al. 2018). Fortunately, an erroneous magnitude for *k*_*s*_ does not affect the value of *s*^o^, the parameter most commonly being sought because of its relevance to the prediction of protein shape from hydrodynamic parameters (Garcia de la Torre et al. 2000; Garcia de la Torre and Harding, 2013), although *k*_s_, if measured correctly, is itself useful in the delineation of molecular shape. In the event that the extent of boundary spreading is being used to evaluate the translational diffusion coefficient *D* the magnitude of *k*_*s*_ also becomes important because of its use to make quantitative allowance for the effects of boundary sharpening arising from the linear negative *s*–*c* dependence exhibited by globular proteins (Fujita 1956, 1959; Baldwin 1957; Van Holde 1960; Scott et al. 2015; Winzor and Scott 2018; Chaturvedi et al. 2018). In that regard, the recommendation that use of the commonly used SEDFIT program for determining protein molar mass from sedimentation velocity distributions be confined to the analysis of experiments with low loading concentrations (Schuck 2005) reflected the omission of any allowance for this boundary sharpening effect at that stage — a point now emphasized in the recent study dealing with the consequences of hydrodynamic nonideality (Chaturvedi et al. 2018).

### Elimination of the radial position of the meniscus *r*_m_ as a variable in data analysis

Another point to note is the universal reliance of current procedures upon the equivalent of Eq. (5) to eliminate time-dependence of the abscissa parameter in the derived sedimentation coefficient distribution. Such transformation of radial distance *r* at time *t* into a sedimentation coefficient calculated as ln (*r*/*r*_*m*_)/(ω^2^*t*) is contingent upon accurate location of the air-liquid meniscus position *r*_*m*_. As noted above, a concern in the analysis of interferometric records of sedimentation velocity distributions is therefore the current lack of adequate definition of *r*_*m*_, a fundamental parameter in specifying the initial conditions in a sedimentation velocity experiment, namely *c* = *c*_*o*_ for *r* ≥ *r*_*m*_. As demonstrated in Fig. 3b, the elucidation of *r*_*m*_ from the time-dependence of ln *r*_*b*_ underestimates *r*_*m*_ slightly (5.797 cf 5.800 cm) as the result of assuming linearity of a dependence that is undetectably curvilinear. Indeed, this error in the value of *r*_*m*_ is responsible for the slight overestimation of $\overline{s}$ values and hence underestimation of *k*_*s*_ that was observed (Patel et al. 2018) in applications of the best-available procedure (Kegeles and Gutter 1951) to numerically simulated sedimentation velocity distributions for ovalbumin and equine γ-globulin. In the standard SEDFIT and SEDANAL programs, the inclusion of *r*_*m*_ as an additional parameter allows further minimization of the uncertainty inherent in the returned sedimentation coefficient, but its assumed constancy clearly disregards the continual increase in *s* as the result of radial dilution. Although the returned magnitude of *r*_*m*_ is therefore an underestimate, the stance has been taken (Schuck 2000, 2005; Brown et al. 2009) that it affords a more reliable definition of the air–liquid meniscus position than any direct attempt at location from examination of the poorly resolved meniscus region.

One possible solution to this dilemma would entail use of the decline in plateau concentration as the result of radial dilution, *c*_*p*_ = (*r*_*m*_/*r*_*b*_)^2^*c*_*o*_, to determine the meniscus position from the slope of the dependence of the square root of the concentration ratio, (*c*_*p*_/*c*_*o*_)^1/2^, upon the reciprocal of boundary position *r*_*b*_. Alternatively, because specification of the boundary position is central to current sedimentation coefficient measurements, the inclusion of absorption – or better – schlieren (see below) optical records of the sedimentation velocity distributions in the data files could well lead to considerable improvement over the Rayleigh system in providing a more definitive experimental estimate of *r*_*m*_. Elimination of a model-dependent estimate of *r*_*m*_ would be beneficial because of (a) a decrease in the number of curve-fitting parameters to be evaluated by the analysis, and (b) removal of the iteration involved in estimating the lower limit of the radial distance range over which the Lamm equation is being integrated in the *c*_*NI*_(*s*^0^) procedure (Chaturvedi et al. 2018). In principle, the only remaining deficiency of the analysis would then be the approximation involved in identifying the printout value of the integral over time of the angular velocity squared (∫ω^2^d*t*) with the corresponding parameter since attainment of the specified initial state (*c* = *c*_*o*_ for *r* ≥ *r*_*m*_): an experimental study has placed that effective time correction factor at about 2 min (Besong et al. 2012)

In view of the complexity of the refined and potentially more accurate procedure for defining *s*-*c* dependence (Chaturvedi et al. 2018), use of the standard *c*(*s*) (Li et al. 2017) and g*(*s*) (Sun et al. 2004) methods is likely to continue. However, the concentration assigned to the sedimentation coefficient from a given run needs to be calculated as $\overline{c}$, and not identified with the loading concentration *c*_*o*_. To that end it is recommended that if *k*_s_ is to be utilised in a quantitative way the analysis be confined to sedimentation velocity distributions that are fully resolved from the air-liquid meniscus and retain a clearly defined plateau concentration *c*_*p*_ to allow the estimation of $\overline{c}$ as the mean of *c*_*p*_ values for the first and final distributions used for the analysis (Patel et al. 2018).

## Perspectives

This retrospective investigation of the problem has established that attempts to improve the characterization of *s*–*c* dependence for globular proteins by the development of quantitative expressions in terms of the loading concentration in sedimentation velocity experiments (Alberty 1954; Trautman et al. 1954; Fujita 1956; Baldwin 1957) were all doomed to failure because of experimental limitations. Specifically, the predicted continual increase in sedimentation coefficient in response to an ever-decreasing plateau concentration as the result of radial dilution was too small for experimental detection in the schlieren optical records of sedimentation velocity distributions then available. Unfortunately, the situation has worsened since then because of greater difficulties in locating the position of the air–liquid meniscus (*r*_*m*_) in current Rayleigh interference optical records — a difficulty that has been side-lined by regarding *r*_*m*_ as an additional parameter to be determined by analysis of the sedimentation velocity distributions (Schuck 2000; Brown et al. 2009). However, that *c*(*s*) approach leads to an underestimate of *r*_*m*_ because of its determination on the basis of a single value of the sedimentation coefficient throughout the experiment; and hence decreases even further the reliability of the time-dependent sedimentation coefficients, *s*^∗^(*t*) = [ln(*r*_*b*_/*r*_*m*_)]/(ω^2^*t*), that play a pivotal role in the attempts to incorporate directly the effects of radial dilution into the quantification of linear *s*–*c* dependence. That quantification is therefore most simply achieved by reverting to the original approach (Kegeles and Gutter 1951) in which the sedimentation coefficient obtained without allowance for the effects of radial dilution [by standard *c*(*s*), g*(*s*) or *G*(*s*) analysis] is regarded as an average parameter ($\overline{s}$) that pertains to the corresponding mean plateau concentration ($\overline{c}$) over the range of sedimentation velocity distributions used for the determination of $\overline{s}$ (Patel et al. 2018). Ironically, the most accurate delineation of *s-c* dependence would entail reversion to the currently discarded practice of determining sedimentation coefficients from the time-dependence of ln *r*_*b*_ because of its avoidance of an absolute time scale or knowledge of *r*_*m*_. Any indecision about the precise location of *r*_*b*_ is removed by its calculation as the square root of the second moment of the boundary at time *t* (Goldberg 1953).

In other words, the developments in computer technology that have led to vast advances in procedures for data collection and manipulation have not yet facilitated the quest for more accurate quantitative characterization of linear *s*-*c* dependence. The barrier to solving that problem has been an inability to detect, let alone quantify, the variation in sedimentation coefficient with radial distance across a concentration distribution at time *t *— a barrier that plagued ultracentrifuge chemists in the 1950s and continues to do so seven decades later for want of an optical system that yields registration of concentration distributions in which the radial position of the air-solvent meniscus can be defined with the precision required for the application of theory developed six decades ago. Progress towards alleviating that experimental limitation could well result from incorporation of the schlieren optical system to obtain a third optical record (radial dependence of the solute concentration gradient) in the Beckman XL-I ultracentrifuge. Although not having the precision of Rayleigh interference optics for registering solute concentrations, the schlieren refractometric optical system (a mainstay of the model E but discarded in the XL-I) possesses a major advantage over both the Rayleigh and absorption optical systems — namely accuracy with which the air-solvent meniscus position *r*_m_ can be ascertained. Simultaneous registration of schlieren and Rayleigh records would lead to removal of *r*_m_ as a curve-fitting parameter in the analysis whilst preserving the accuracy of concentration registration, thereby facilitating the determination of *s*-*c* profiles from single measurements with sufficient accuracy. Further, provision of a modern on-line schlieren system but based on the sound optical principles of the last century (Lloyd 1974) would also facilitate optical registation of concentration profiles well beyond existing instrumental limit of about 50 g/L for current interference optics. Concentrations well in excess of 100 g/L are relevant to current delivery of many protein and glycan based therapeutics. Such provision would hopefully stimulate a greater understanding of the theoretical dependence of both sedimentation and diffusion processes and associative/aggregative phenomena in the analytical ultracentrifuge across the complete range of medically and industrially relevant concentrations up to 450 g/L (Sønderby et al. 2018), an order of magnitude greater than the current capability of the optical systems on analytical ultracentrifuges.
